# Feasibility of Bispectral Index-Guided Propofol Infusion for Flexible Bronchoscopy Sedation: A Randomized Controlled Trial

**DOI:** 10.1371/journal.pone.0027769

**Published:** 2011-11-23

**Authors:** Yu-Lun Lo, Ting-Yu Lin, Yueh-Fu Fang, Tsai-Yu Wang, Hao-Cheng Chen, Chun-Liang Chou, Fu-Tsai Chung, Chih-Hsi Kuo, Po-Hao Feng, Chien-Ying Liu, Han-Pin Kuo

**Affiliations:** Department of Thoracic Medicine, Chang Gung Memorial Hospital, Chang Gung University, School of Medicine, Taipei, Taiwan; Emory University, United States of America

## Abstract

**Objectives:**

There are safety issues associated with propofol use for flexible bronchoscopy (FB). The bispectral index (BIS) correlates well with the level of consciousness. The aim of this study was to show that BIS-guided propofol infusion is safe and may provide better sedation, benefiting the patients and bronchoscopists.

**Methods:**

After administering alfentanil bolus, 500 patients were randomized to either propofol infusion titrated to a BIS level of 65-75 (study group) or incremental midazolam bolus based on clinical judgment to achieve moderate sedation. The primary endpoint was safety, while the secondary endpoints were recovery time, patient tolerance, and cooperation.

**Results:**

The proportion of patients with hypoxemia or hypotensive events were not different in the 2 groups (study vs. control groups: 39.9% vs. 35.7%, *p* = 0.340; 7.4% vs. 4.4%, *p* = 0.159, respectively). The mean lowest blood pressure was lower in the study group. Logistic regression revealed male gender, higher American Society of Anesthesiologists physical status, and electrocautery were associated with hypoxemia, whereas lower propofol dose for induction was associated with hypotension in the study group. The study group had better global tolerance (*p*<0.001), less procedural interference by movement or cough (13.6% vs. 36.1%, *p*<0.001; 30.0% vs. 44.2%, *p* = 0.001, respectively), and shorter time to orientation and ambulation (11.7±10.2 min vs. 29.7±26.8 min, *p*<0.001; 30.0±18.2 min vs. 55.7±40.6 min, *p*<0.001, respectively) compared to the control group.

**Conclusions:**

BIS-guided propofol infusion combined with alfentanil for FB sedation provides excellent patient tolerance, with fast recovery and less procedure interference.

**Trial Registration:**

ClinicalTrials. gov NCT00789815

## Introduction

Patients undergoing flexible bronchoscopy (FB) experience procedure-related symptoms [1]. Benzodiazepines (i.e., midazolam) plus an opioid is the most common combination used to improve patient tolerance and satisfaction [2], [3]. Current guidelines recommend incremental midazolam sedation to all patients undergoing FB, except when there are contraindications [4]. The required dose varies, and its prolonged effect delays patient recovery [5]. Moreover, a bolus of midazolam is often administered when patients suffer from procedure related discomfort that interferes with bronchoscopic procedures.

With the advances in sedative drugs and monitors, several FB sedative protocols have been recently investigated. Sedation with intermittent propofol (2,6-diisopropylphenol) bolus has shown to provide good tolerance and fast recovery in patients undergoing FB [6], [7], [8]. Because of its short time to peak concentration (2 min) and fast redistribution and clearance, propofol is suitable and easily titratable to maintain steady plasma concentrations with continuous infusion [9], [10]. Adding opioids may provide antitussive effects and modify the pharmacokinetics of propofol, which reduces the required propofol dose [9], [11], [12]. Alfentanil is ideal for FB because of its fast onset and short duration [13], [14], [15]. However, controversy about combining propofol and opioids persists because of the risk of over-sedation and cardiopulmonary depression [16], [17].

The bispectral index (BIS) is a non-invasive and objective indicator of the depth of anesthesia. Its algorithm processes patients' electroencephalography (EEG) and electromyography (EMG) data and computes an index from 0 (isoelectric EEG) to 100 (fully awake). Good correlations between propofol drug concentration, sedative score, and BIS level have been shown [18], [19], [20], [21], and BIS-guided propofol bolus maintaining a BIS index between 70 and 85 has been studied in simple FB procedures [7]. Larger studies are needed to evaluate the feasibility of BIS-guided propofol sedation in FB. Furthermore, the increasing use of interventional FB requires more objective and efficient methods of sedation delivery[22]. Therefore, we designed a sedative protocol combining the advantages of propofol infusion and alfentanil, using BIS monitoring to maintain a level of 65 to 75, in order to provide efficient sedation for patients undergoing complex and long duration procedures.

This study hypothesized that BIS-guided propofol infusion is as safe as the current standard method of clinically-judged midazolam sedation, and may even provide better FB sedation.

## Methods

This prospective, randomized study was conducted in a tertiary medical center. The institutional review board of the Chang Gung Memorial Hospital approved the study protocol (No. 97-0257B) and the enrolled patients provided written informed consent. The protocol for this trial and supporting CONSORT checklist are available as supporting information; see Checklist S1 and Protocol S1. Patients undergoing elective FB and sedation were screened for enrollment. The exclusion criteria were age <18 years, American Society of Anesthesiologists (ASA) physical status classification IV or V, neurologic disorders or other conditions contributing to difficulty in assessing response, forced expiratory vital capacity (FVC) <15 mL/kg body weight, forced expiratory volume in one second (FEV1) <1000 mL or FEV1/FVC <35%, or a Mallampati score of 4. Patients with a known history of allergy to the study drugs, eggs, soybeans or sulfite products, or those with glaucoma were also excluded. The enrolled patients were randomized by an investigator according to a predetermined computer code.

### Sample size

A preliminary study following the patient preparation, premedication and sedative protocol was performed before this trial. Sixteen patients undergoing FB in both groups were analyzed. The proportion of patients recorded with at least one episode of desaturation (oxygen saturation <90% with any duration) in each group was 0.31 and 0.19, respectively. A difference of >12% in the percent of patients experiencing desaturation would be considered clinically important [23]. A sample size of 250 per group was selected to provide 90% power to detect such a difference using a two sided 5% level of significance.

### Patient preparation

Blood pressure was monitored using an automated pressure cuff, and heart rate and rhythm were monitored by three-lead electrocardiography. A peripheral pulse oximeter was used to monitor oxyhemoglobin saturation (SpO_2_) while a nasal cannula delivered 2 L/min of oxygen to the patient. An intravenous catheter was placed in the forearm for drug administration. A disposable BIS Quatro Sensor (Aspect Medical System Inc, Newton, MA, USA) was applied to the forehead of patients in the study group. The sensor was connected to the A-2000 XP BIS monitor (Version 3.11, Aspect Medical Systems, Inc.). A BIS value was displayed once all impedances were acceptable. The smoothening time was set at 15 s. All parameters were monitored continuously except for blood pressure, which was recorded every 3 min.

Topical anesthesia with nebulized lidocaine and the “spray as you go” technique was used for local anesthesia, as described elsewhere [24]. An experienced bronchoscopist assisted by a well-trained technician performed the FB. One investigator responsible for sedation techniques monitored cardiopulmonary functions to determine the need for interventions, such as increasing the oxygen delivery to 6 L/min to maintain oxygen saturation above 90%, jaw support, manual assisted ventilation with an ambubag for persistent desaturation, to maintain adequate airways or fluid resuscitation, and leg elevation for hypotension.

All bronchoscopists and investigators were qualified for intensive and critical care and advanced cardiac life support. They were also familiar with the sedation drugs used for FB sedation [4]. The resuscitation equipment and drugs were readily available.

### Sedation protocol

In the study group, induction was performed using alfentanil (1∶10 dilution, 4-5 µg/kg bolus) following an initial administration of 0.5 mg/kg intravenous propofol bolus. The dose of propofol was then carefully titrated by administering 10–20 mg boluses until the BIS index reached 70. The duration between boluses was 20 s. The propofol infusion (3–12 mg/kg/h) was then administered using a syringe pump (Injectomat Agilia, Fresenius Kabi, France) to maintain the BIS index between 65 and 75. If cough-related BIS elevation occurred, cough management was performed as described below, rather than increasing the dose of propofol.

In the control group, induction was performed using alfentanil (4–5 µg/kg bolus) following a 2 mg midazolam bolus. According to official guidelines of FB [4], if the patient was not well-sedated after 2 min, midazolam bolus was repeated in increments of 1 mg/min until moderate sedation (purposeful response to verbal or tactile stimulation) was achieved [4], [25]. For maintenance, 1 mg/min midazolam boluses were administered based on clinical judgment to achieve moderate sedation or if persistent patient movement or severe cough interfered with the procedure.

In both groups, if the bronchoscopist deemed that persistent cough interfered with the procedure, oral secretions were suctioned and/or 2 mL 1% lidocaine was instilled via the bronchoscope. If cough persisted and the management proved insufficient, an alfentanil bolus (1–2 µg/kg) was administered for every 15 min. After the procedure, the patients were sent to the recovery room and monitored continuously until full recovery.

### Assessment

Adverse events were evaluated as the proportion of patients with at least 1 event of hypotension (systolic blood pressure [SBP] <90 mm Hg or mean arterial blood pressure [MAP] <60 mm Hg of any duration) or hypoxemia (SpO_2_ <90% of any duration) during FB. The lowest SpO_2_ and blood pressure values were also recorded.

Bronchoscopists assessed patient cooperation in the following manner. “Procedural interference by patient movement” meant that the bronchoscopist had to pause the procedure temporarily and the assistant needed to physically restrain the irritated patient. “Procedural interference by cough” meant that the bronchoscopist had to pause the procedure temporarily and additional xylocaine spray and/or alfentanil had to be administered to stop the coughing.

Recovery was evaluated by time to orientation and time to ambulation. Time to orientation was defined as the duration between finishing FB and the point when the patients could spontaneously open their eyes, recall their date of birth, and correctly perform the finger-to-nose test [6]. Time to ambulation was defined as the duration between finishing FB and the point when the patients could walk without assistance. After recovery, the patients answered a questionnaire regarding procedure-related symptoms, including nebulized anesthetic inhalation, scope insertion, cough, dyspnea, pain, and global tolerance to the entire procedure on a 10-point verbal analogue scale (VAS, 0: no bother, 10: worst intolerable) as recorded by an investigator blinded to their groupings.

The sedative doses and the duration of induction as well as of the procedures were recorded. Induction time was defined as the duration between alfentanil administration and the point when the desired sedation level was attained. FB duration was defined as the time period between the insertion and removal of the bronchoscope. On the third to fifth day post-FB, the general condition of the patientswas followed up, either by outpatient visits or by telephone correspondence.

### Statistical analysis

Age, body weight, drug doses, and duration were presented as mean ± standard deviation (SD) and analyzed by the Student's t-test. The VAS was presented as accumulative percentage in each group, and analyzed by the Mann-Whitney U test. The chi-square test was used to analyze gender, physical status, indications, and procedures, as well as adverse events and patient cooperation. Univariate Student's t-test and chi-square tests where appropriate were performed to determine which factors including patient characteristics, indications of bronchoscopy, procedures performed, sedative dosing of induction/total procedures, and duration of induction/total procedure were significantly associated with hypoxemia or hypotension (MAP <60 mm Hg or SBP <90 mm Hg) in the study group. All factors significant in univariate analysis were further analyzed by the multivariate logistic modeling. Statistical significance was set at *p*<0.05. Statistical analyses were performed using the Statistical Package for Social Sciences version 13 (SPSS Inc., Chicago, IL, USA).

## Results

**Figure 1:**
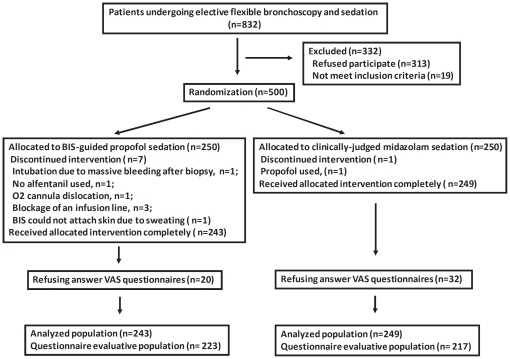
Patient disposition. BIS, bispectral index; VAS, verbal analogue scale.

From April 2008 to September 2009, 500 patients undergoing elective FB were randomized (Figure 1), and 243 and 249 patients completed the intervention in the study and control groups, respectively. Both groups had comparable basic characteristics, indications, and FB procedures (Table S1). More than 75% of patients were outpatients, and 40% were ASA class 3. More than 70% patients underwent at least two procedures. The major indications for FB were lung or mediastinal nodules/masses, and the most common procedure was biopsy (68.3% of all patients) of the lungs or mediastinum.

**Figure 2:**
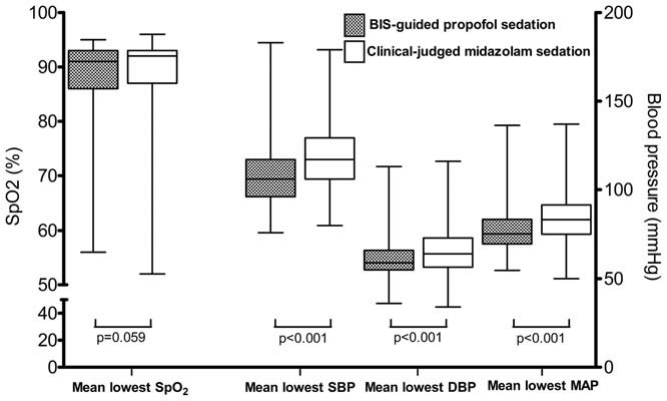
The mean lowest oxygen saturation and blood pressure in both groups. Boxes represent median and inter-quartile range; whiskers represent range. BIS, bispectral index; SpO_2_, oxyhemoglobin saturation; MAP, mean arterial blood pressure; SBP, systolic blood pressure; DBP, diastolic blood pressure.

The mean duration of FB was 25 min, and the proportion of patients with hypotension or hypoxemia, as well as the lowest SpO_2_ value was similar in both groups (Table 1 and Figure 2). The mean lowest blood pressure was significantly lower in the study group, and all recovered spontaneously or following proper management. Vasopressor administration was not required; mortality was also not recorded. In the study group, 1 patient was intubated and mechanical ventilation was performed because of massive bleeding after bronchial biopsy, and 1 patient developed a pneumothorax that required chest tube drainage. In the control group, 1 patient was administered flumazenial for a poor respiratory pattern during recovery and was admitted to the intensive care unit. These patients recovered without sequelae. On following up after 3–5 days, no significant serious complications were noted in either group. Logistic regression revealed that male gender, higher ASA physical status, and electrocautery were associated with hypoxemia and lower induction doses of propofol were associated with hypotension in the study group (Table 2).

**Table 1:** Bronchoscopy and sedative outcomes.

	BIS-guided Propofol Sedation (*n* = 243)	Clinically-judged Midazolam Sedation(*n* = 249)	*p* value
**Induction**			
P/M dose, mg	54.5 (16.4)	3.8 (2.5)	
A dose, µg	295.0 (54.8)	285.2 (56.4)	0.052
Induction time,* min	3.4 (1.6)	5.3 (3.1)	<0.001
**Total procedures**			
P/M dose, mg	198.6 (102.7)	6.8 (3.9)	
A dose, µg	325.4 (101.7)	350.3 (117.1)	0.012
Mean BIS level	70.8 (6.1)		
Procedure time,† min	25.8 (15.2)	25.5 (16.2)	0.810
**Recovery time, min**			
Time to orientation‡	11.5 (10.2)	30.0 (26.8)	<0.001
Time to ambulation¶	30.0 (18.1)	55.7 (40.6)	<0.001
**Safety** #			
SpO_2_ <90%	97 (39.9)	89 (35.7)	0.340
MAP <60 mm Hg	11 (4.5)	4 (1.6)	0.060
SBP <90 mm Hg	18 (7.4)	11 (4.4)	0.159

Data is presented as mean (SD) unless otherwise indicated.

Abbreviations: P, propofol; M, midazolam; A, alfentanil; SpO2: oxyhemoglobin saturation; MAP: mean arterial blood pressure; SBP: systolic blood pressure.

*From alfentanil administration to BIS level 70 in the study group, or conscious sedation in the control group.

†From insertion of bronchoscope to its removal.

‡Patients could open eyes spontaneously, correctly recall date of birth, and perform finger-nose test.

¶Patients could walk without assistance.

# The number of patients with at least one event of hypoxemia or hypotension during the entire procedure (percent).

**Table 2:** Univariate and logistic regression analysis of factors associated with hypoxemia or hypotension in patients under BIS-guided propofol sedation.

Factors	OR	95% CI	*p* value
**Univariate analysis** *			
**Hypoxemia** †			
Male vs. female	1.74	1.02–2.96	0.041
ASA physical status 3 vs. 1/2	2.30	1.36–3.90	0.002
Electrocautery	5.10	1.36–19.00	0.015
**Hypotension** ‡			
Endobronchial obstruction	5.33	1.50–18.89	0.001
Induction dose of propofol	0.96	0.92–0.99	0.019
Electrocautery	5.94	1.65–21.44	0.006
**Logistic regression** ¶			
**Hypoxemia** †			
Male vs. female	1.75	1.01–3.04	0.047
ASA physical status 3 vs. 1/2	2.20	1.29–3.77	0.004
Electrocautery	5.16	1.34–19.91	0.017
**Hypotension** ‡			
Induction dose of propofol-	0.96	0.93–1.00	0.041

Abbreviations: OR, odds ratio; CI, confidence interval; ASA, American Society of Anesthesiologists.

*Patient, procedure, sedative factors were analyzed by univariate Student's t-test and chi-square tests where appropriate.

†Oxyhemoglobin saturation <90% for any duration.

‡Mean arterial pressure <60 mm Hg or systolic blood pressure <90 mm Hg for any duration.

¶Factors significant in the univariant analysis were analyzed and adjusted together by the multivariate logistic modeling.

**Figure 3:**
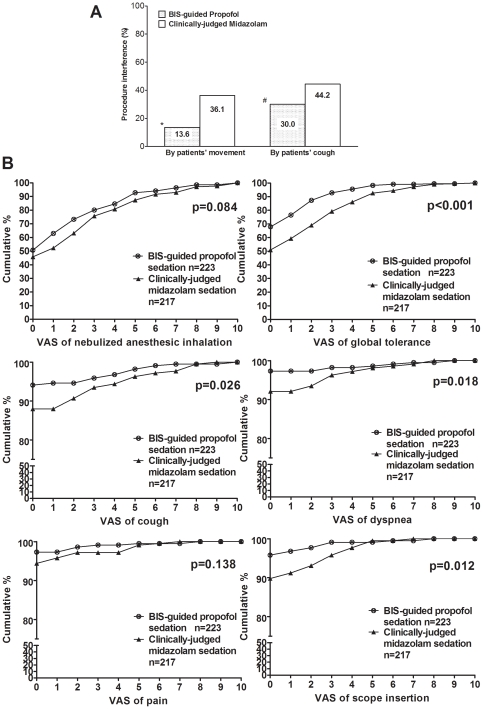
Patient cooperation was accessed by procedural interference during bronchoscopy (A) and patient tolerance of procedure-related symptoms and global tolerance during bronchoscopy was accessed by verbal analogue scale (VAS) (B). A: Interference by patient movement: The bronchoscopist had to temporarily pause the procedure and the assistants had to restrain the patient. Interference by patient coughing: The bronchoscopist had to pause the procedure temporarily and additional xylocaine spray and/or alfentanil had to be administered to stop the coughing. *p<0.001 vs. clinically-judged midazolam; #*p* = 0.001 vs. clinically-judged midazolam. B: After recovery, patient tolerance was evaluated by VAS (0: no bother, 10: worst intolerable). Data are presented as accumulative percentage of VAS in each group. A lower VAS score indicates better tolerance.

Patients in the study group achieved the desired level of sedation more rapidly than those in the control group did (Table 1). Procedure time was similar in both groups, and the mean BIS level was 70.8 in the study group. Procedural interference by patient movement or cough was significantly less in the study group (Figure 3A), and the total dose of alfentanil was higher in the control group because more supplemental doses were required due to a higher incidence of severe coughing. Patients in the study group recovered their orientation faster and were able to walk without assistance in a shorter time. They also showed significantly better tolerance for bronchoscope insertion, cough, and dyspnea during FB, as well as global tolerance for the whole procedure, as scored by VAS (Figure 3B).

## Discussion

To the best of our knowledge, this is the first prospective randomized study of alfentanil in combination with continuous propofol infusion titrated to maintain a BIS level of 65–75 for FB sedation. This study revealed that BIS-guided propofol infusion is as safe as the current standard method of clinically-judged midazolam sedation, in terms of the proportion of patients experiencing hypoxemia and hypotension. The mean lowest blood pressure was lower in the study group compared to the control group, and all patients recovered spontaneously or after proper management. This study proves that BIS-guided propofol sedation provides better tolerance and faster recovery to patients undergoing FB, and provides faster induction and less procedural interference for the bronchoscopists. These findings are clinically valuable, especially for complicated and time-consuming interventional bronchoscopic procedures.

The BIS algorithm was developed from a large database of patients or volunteers receiving various anesthetic regimens [19]. However, it has not been applied routinely during FB, and the optimal BIS level for FB sedation is not yet established. Grass et al. [19] reported that 95% of healthy volunteers under propofol sedation become amnesic at a mean BIS level of 77 (95% CI 72–83), and 50% lose their response to verbal commands at a mean BIS level of 63 (95% CI 62–65). Bauer et al. [26], in another propofol sedation study of patients undergoing surgery, reported that patients could still move in response to pain stimulation when the mean BIS level was 69. In another study using BIS monitoring during gastrointestinal endoscopy sedation, Bower et al. [20] reported that patients responded to mild prodding, which is considered the level of moderate sedation, at a median BIS level of 67.5 (range, 54–97). Thus, a BIS level of 65–75 was set for FB sedation, and a BIS level of 70 was set for induction in this protocol to achieve patients that are amnesic but still with reflex responsiveness to noxious stimulation at a relatively level of sedation. If cough-related BIS elevation occurred, coughing was first treated as mentioned in our method., instead of introducing propofol.

Recently, Clark et al. [7] compared administration of single drug bolus of propofol to midazolam while maintaining the BIS level at 70–85 for a small number of patients undergoing simple FB with better ASA class. Patients receiving propofol showed better global tolerance, but no difference in the perception of coughing and there was no difference in the bronchoscopists' assessment, comparing to the control patients. Stolz et al. [8] reported that the discomfort score and safety profiles of patients receiving sedation for FB with propofol bolus based on clinical judgment were similar to those with sedation induced using combined midazolam and hydrocodone, although coughing was more severe with propofol treatment. Compared to these studies, the FB procedures employed in the current study were more complicated and time-consuming, but the study revealed that BIS-guided propofol infusion with alfentanil administration provides additional benefits for the bronchoscopists (less procedural interference) and patients (less discomfort from scope insertion, dyspnea, and cough). Compared to intermittent administration of boluses, which may result in fluctuating plasma concentrations and risk of over- or under-sedation, continuous propofol infusion provides a more steady plasma concentration that can be titrated within the therapeutic window [9], [10], [27]. Adding alfentanil can modify the pharmacokinetic property of propofol, reduce the required dose of propofol, and facilitate faster recovery with less cardiovascular depression [9], [11], [12]. A BIS monitor provides a trend of EEG change during a short processing time (usually 15 s) and gives the feedback of conscious level changing fromthe patient responsiveness to the procedures and drugs, thereby helping sedative drug adjustment. Thus, a BIS-guided propofol infusion can provide a steadier drug concentration and effect, as well as allow instant and individualized titration. This may explain why BIS-guided sedation was better tolerated and had reduced irritated movements by patients, without increased adverse effects in this study. Despite higher doses of propofol, and more complicated and longer procedures, the incidence of hypotension was similar to that reported by Clark et al. [7] (3.7–7.4% vs. 4.7%) and that of hypoxemia (39.9%) was consistent with previous reports (34.9% and 32%, respectively) [7], [8].

Logistic regression revealed that electrocautery, ASA class 3, and male gender were associated with hypoxemia in the study group. Electrocautery often generates tissue debris and blooding that contribute to ventilation/perfusion mismatch in patients with endobronchial lesions during prolonged procedures. Patients with higher ASA physical status may have relatively inadequate cardiopulmonary function. In the current study, men received more alfentanil during induction than women (310.8 vs. 271.1 µg, *p*<0.001) because of higher mean weight (64.4 vs. 56.0 kg, *p*<0.001), and had a higher incidence of hypoxemia during induction (20% vs. 9.2%, *p* = 0.023). The higher amount of alfentanil administered to men may be a contributing factor to the increase in the incidence of hypoxemia in men. Another explanation may be the gender-based difference in the pharmacokinetic properties of alfentanil or propofol [28]. However, this study was not designed to investigate such parameters. For male patients with ASA physical status 3 or those with endobronchial obstructions where electrocautery is indicated, propofol infusion should be performed with caution.

Logistic regression also revealed that a lower induction dose of propofol was associated with hypotension. Patients who required less propofol for induction may be more sensitive to propofol. This may be explained by different interpatient susceptibilities to propofol due to the difference in cardiac output, hepatic perfusion, body fat, and haplotype differences in metabolic genes, and requires to be studied further[29]. This is a valuable hint for clinical practice. If induction is achieved rapidly, sedative procedures should be performed with caution. The BIS has been used to monitor propofol titration for sedation of various procedures [7], [12], [20], [21]; however, there are other aspects of FB sedation that require further study. Besides the cost-effectiveness of the additional equipment and personnel, the regimen of opioid administration for induction as well as the procedure, the optimal BIS level for FB sedation, and the propofol infusion profile for better pharmacokinetic control (e.g., targeted control infusion) require further investigation to improve FB sedation.

This study has some limitations that should be considered. First, the investigators and bronchoscopists were not blinded to the sedation procedures. Because the aim of this study was to compare the current standard practice of clinically-judged incremental midazolam sedation to BIS-guided propofol continuous infusion, it is difficult to use completely blinded conditions because of maior difference in the protocols. The bronchoscopist can distinguish the protocol by observing the actions of the investigators while patients without irritated motion but BIS level reaching to the criteria for drug titration. Nonetheless, the primary endpoints were hypoxemia and hypotension events, which were recorded objectively. Second, patients with a history of FB were not excluded. Many patients indications for interventional bronchoscopy often undergo FB first for lesion site evaluation or for pathologic confirmation. Such previous experience with FB may affect a patient's judgment regarding their tolerance of FB. However, the numbers of patients with previous FB experience in the two groups were similar (data not shown); therefore, this should not influence data interpretation.

In conclusion, the current study showed that BIS-guided propofol infusion combined with alfentanil is feasible and safe. It provides excellent tolerance and fast recovery for the patients undergoing FB. It also facilitates the performance of procedures and reduces patient interference. Further studies on induction, BIS level, and propofol infusion profile for FB sedation as well as cost-effectiveness of BIS guided propofol infusion are warranted to improve the safety and quality of FB sedation.

## Supporting Information

Table S1 Patient characteristics, indications for flexible bronchoscopy, and procedures performed.
(DOC)

Checklist S1 CONSORT Checklist.
(DOC)

Protocol S1 Trial Protocol.
(DOC)

## References

[1] Diette GB, White P, Terry P, Jenckes M, Wise RA (1998). Quality assessment through patient self-report of symptoms prefiberoptic and postfiberoptic bronchoscopy.. Chest.

[2] Pickles J, Jeffrey M, Datta A, Jeffrey AA (2003). Is preparation for bronchoscopy optimal?. Eur Respir J.

[3] Matot I, Kramer MR (2000). Sedation in outpatient bronchoscopy.. Respir Med.

[4] British Thoracic Society Bronchoscopy Guidelines Committee (2001). British Thoracic Society guidelines on diagnostic flexible bronchoscopy.. Thorax.

[5] Williams TJ, Bowie PE (1999). Midazolam sedation to produce complete amnesia for bronchoscopy: 2 years' experience at a district general hospital.. Respir Med.

[6] Crawford M, Pollock J, Anderson K, Glavin RJ, MacIntyre D (1993). Comparison of midazolam with propofol for sedation in outpatient bronchoscopy.. Br J Anaesth.

[7] Clark G, Licker M, Younossian AB, Soccal PM, Frey JG (2009). Titrated sedation with propofol or midazolam for flexible bronchoscopy: a randomised trial.. Eur Respir J.

[8] Stolz D, Kurer G, Meyer A, Chhajed PN, Pflimlin E (2009). Propofol versus combined sedation in flexible bronchoscopy: a randomised non-inferiority trial.. Eur Respir J.

[9] Lichtenbelt BJ, Mertens M, Vuyk J (2004). Strategies to optimise propofol-opioid anaesthesia.. Clin Pharmacokinet.

[10] Gepts E (1998). Pharmacokinetic concepts for TCI anaesthesia.. Anaesthesia.

[11] Lysakowski C, Dumont L, Pellegrini M, Clergue F, Tassonyi E (2001). Effects of fentanyl, alfentanil, remifentanil and sufentanil on loss of consciousness and bispectral index during propofol induction of anaesthesia.. Br J Anaesth.

[12] Gan TJ, Glass PS, Windsor A, Payne F, Rosow C (1997). Bispectral index monitoring allows faster emergence and improved recovery from propofol, alfentanil, and nitrous oxide anesthesia. BIS Utility Study Group.. Anesthesiology.

[13] Watts MR, Geraghty R, Moore A, Saunders J, Swift CG (2005). Premedication for bronchoscopy in older patients: a double-blind comparison of two regimens.. Respir Med.

[14] Hwang J, Jeon Y, Park HP, Lim YJ, Oh YS (2005). Comparison of alfetanil and ketamine in combination with propofol for patient-controlled sedation during fiberoptic bronchoscopy.. Acta Anaesthesiol Scand.

[15] Houghton CM, Raghuram A, Sullivan PJ, O'Driscoll R (2004). Pre-medication for bronchoscopy: a randomised double blind trial comparing alfentanil with midazolam.. Respir Med.

[16] Graber RG (1999). Propofol in the endoscopy suite: an anesthesiologist's perspective.. Gastrointest Endosc.

[17] Yoon HI, Kim JH, Lee JH, Park S, Lee CT (2011). Comparison of propofol and the combination of propofol and alfentanil during bronchoscopy: a randomized study.. Acta Anaesthesiol Scand.

[18] Iselin-Chaves IA, Flaishon R, Sebel PS, Howell S, Gan TJ (1998). The effect of the interaction of propofol and alfentanil on recall, loss of consciousness, and the Bispectral Index.. Anesth Analg.

[19] Glass PS, Bloom M, Kearse L, Rosow C, Sebel P (1997). Bispectral analysis measures sedation and memory effects of propofol, midazolam, isoflurane, and alfentanil in healthy volunteers.. Anesthesiology.

[20] Bower AL, Ripepi A, Dilger J, Boparai N, Brody FJ (2000). Bispectral index monitoring of sedation during endoscopy.. Gastrointest Endosc.

[21] Miner JR, Biros MH, Seigel T, Ross K (2005). The utility of the bispectral index in procedural sedation with propofol in the emergency department.. Acad Emerg Med.

[22] Kuo CH, Chen HC, Chung FT, Lo YL, Lee KY (2011). Diagnostic Value of EBUS-TBNA for Lung Cancer with Non-Enlarged Lymph Nodes: A Study in a Tuberculosis-Endemic Country.. PLoS One.

[23] Clarkson K, Power CK, O'Connell F, Pathmakanthan S, Burke CM (1993). A comparative evaluation of propofol and midazolam as sedative agents in fiberoptic bronchoscopy.. Chest.

[24] Stolz D, Chhajed PN, Leuppi JD, Brutsche M, Pflimlin E (2004). Cough suppression during flexible bronchoscopy using combined sedation with midazolam and hydrocodone: a randomised, double blind, placebo controlled trial.. Thorax.

[25] Force ASoAT (2002). Practice guidelines for sedation and analgesia by non-anesthesiologists.. Anesthesiology.

[26] Vernon JM, Lang E, Sebel PS, Manberg P (1995). Prediction of movement using bispectral electroencephalographic analysis during propofol/alfentanil or isoflurane/alfentanil anesthesia.. Anesth Analg.

[27] Hu C, Horstman DJ, Shafer SL (2005). Variability of target-controlled infusion is less than the variability after bolus injection.. Anesthesiology.

[28] Gan TJ, Glass PS, Sigl J, Sebel P, Payne F (1999). Women emerge from general anesthesia with propofol/alfentanil/nitrous oxide faster than men.. Anesthesiology.

[29] Iohom G, Ni Chonghaile M, O'Brien JK, Cunningham AJ, Fitzgerald DF (2007). An investigation of potential genetic determinants of propofol requirements and recovery from anaesthesia.. Eur J Anaesthesiol.

