# Supplementary material for: Feasibility of Bispectral Index-Guided Propofol Infusion for Flexible Bronchoscopy Sedation: A Randomized Controlled Trial
Source: PLoS One. 2011 Nov 23;6(11):e27769. doi: 10.1371/journal.pone.0027769 (PMC3223212; doi:10.1371/journal.pone.0027769)
Supplement: Table S1 — Patient characteristics, indications for flexible bronchoscopy, and procedures performed. (DOC) [file pone.0027769.s001.doc]

**Table S1. Patient characteristics, indications for flexible bronchoscopy, and procedures performed**

|  | **BIS-guided Propofol Sedation (*n* = 243)** | **Clinically-judged Midazolam Sedation (*n* = 249)** | ***p* value** |
| --- | --- | --- | --- |
| **Patient characteristics** |  |  |  |
| Age, yr | 59.9 ± 13.1 | 61.9 ± 14.7 | 0.109 |
| ASA score <3 | 145 (60.4) | 140 (56.2) | 0.439 |
| Male | 145 (59.7) | 139 (55.8) | 0.388 |
| Outpatient | 190 (78.2) | 183 (73.5) | 0.224 |
| Weight, kg | 61.1 (11.3) | 59.9 (11.4) | 0.240 |
| BMI | 23.3 (3.5) | 23.0 (3.9) | 0.570 |
| **Indication for FB** |  |  |  |
| Lung nodules/mass | 126 (51.9) | 110 (44.2) | 0.088 |
| Mediastinal lymph nodes/mass | 60 (24.7) | 61 (24.5) | 0.960 |
| Lung atelectasis/infiltration | 31 (12.8) | 42 (16.9) | 0.200 |
| Endobronchial obstruction | 14 (5.8) | 22 (8.8) | 0.191 |
| Hemoptysis | 6 (2.5) | 10 (4.0) | 0.333 |
| Chronic cough | 5 (2.1) | 3 (1.2) | 0.455 |
| Other | 1 (0.4) | 1 (0.4) | 0.986 |
| **Procedures** |  |  |  |
| EBUS –TBNA | 61 (25.1) | 60 (24.1) | 0.795 |
| Stent loading/removal | 2 (0.8) | 4 (1.6) | 0.429 |
| Electrocautery | 13 (5.3) | 14 (5.6) | 0.894 |
| Auto-fluorescence bronchoscopy | 39 (16.0) | 26 (10.4) | 0.066 |
| Radial probe EBUS | 138 (56.8) | 142 (57.0) | 0.957 |
| Trans-bronchial lung biopsy | 103 (42.4) | 100 (40.2) | 0.616 |
| Bronchial biopsy | 26 (10.7) | 31 (12.4) | 0.544 |
| Bronchial washing | 139 (57.2) | 129 (51.8) | 0.230 |
| Bronchial brushing | 111 (45.7) | 97 (39.0) | 0.131 |
| Bronchoalveolar lavage | 30 (12.3) | 31 (12.4) | 0.972 |
| TBNA | 1 (0.4) | 0 | 0.311 |
| Simple bronchoscopy | 8 (3.3) | 9 (3.6) | 0.845 |
| **Procedures per patient*** |  |  |  |
| One | 50 (20.6) | 62 (24.9) | 0.253 |
| Two | 44 (18.1) | 49 (19.7) | 0.656 |
| Three | 51 (21.0) | 48 (19.3) | 0.636 |
| Four | 66 (27.2) | 59 (23.7) | 0.377 |
| Five | 24 (9.9) | 22 (8.8) | 0.692 |

Data are presented as mean ± standard deviation or number and percentage in parentheses.

*Excludes simple bronchoscopy.

Abbreviations: ASA, American Society of Anesthesiologists; BMI, body mass index; EBUS, endobronchial ultrasound; TBNA, trans-bronchial needle aspiration.
