# Supplementary material for: Feasibility of Bispectral Index-Guided Propofol Infusion for Flexible Bronchoscopy Sedation: A Randomized Controlled Trial
Source: PLoS One. 2011 Nov 23;6(11):e27769. doi: 10.1371/journal.pone.0027769 (PMC3223212; doi:10.1371/journal.pone.0027769)
Supplement: Protocol S1 — Trial Protocol. (DOC) [file pone.0027769.s003.doc]

**Study protocol**

1. Title

| Sedation for flexible bronchosocpy and real time endobronchial ultrasound: a comparison between bispectral index guidance sedation and conventional sedation. |
| --- |

1. Background and Aims

| It is well known that patients undergoing bronchoscopy could be less suffering and the procedures could be carried on more smoothly if the patients have adequate sedation and analgesia. The preferred sedative and analgesic drugs are Midazolam and opioid, like Alfentanil or Morphine, which were titrated according to physicians’ judgment on patients’ clinical responsiveness. However, due to the pharmacokinetic characteristic of midazolam while used in intravenous injection (onset time 4-6 minutes, effective time 2-4 hours), the effective onset time may be too slow for repeated injection while patients already suffered from the bronchoscopic procedure. It is also noted that when over-sedation occurred the side effects like apnea/hypopnea, hypoxemia, and hypotension could last from dozen minutes to few hours. Although events mentioned above could be handled properly under experienced medical staff, it is still very difficult to predict the oncoming events as the pharmacokinetic effect is variant individually. Ideally, it will be more safe and efficient, during invasive procedure like bronchoscope, if the sedative drug could be onset or vanish fast and the drug effect could be titrated with an objective device directly monitoring the depth of sedation or anesthesia.  Propofol is a short-acting [intravenous](http://en.wikipedia.org/wiki/Intravenous) sedative agent used for the induction of general [anesthesia](http://en.wikipedia.org/wiki/Anesthesia) for children and adults; maintenance of general anesthesia; and [sedation](http://en.wikipedia.org/wiki/Sedation) in medical contexts, such as [intensive care unit](http://en.wikipedia.org/wiki/Intensive_care_unit) (ICU) sedation for intubated, mechanically ventilated adults, and in procedures such as [colonoscopy](http://en.wikipedia.org/wiki/Colonoscopy). Its mechanism of action is uncertain, but it is postulated that its primary effect may be potentiation of the [GABA-A](http://en.wikipedia.org/wiki/GABA_A_receptor) receptor, possibly by slowing the channel closing time. It has a fast onset time (1~2 minutes) but a short working duration (8~10 minutes), which vanished fast after stop administration. Bispectral Index (BIS), an non-invasive neurophysiologic monitor instrument, can transform the EEG and EMG of the patient to a continual numeral, ranging from 0 to 99, which provides a direct and real-time sedative depth monitor. A BIS value of 0 equals EEG silence, near 100 is the expected value in a fully awake adult, and below70 indicated the patient lose explicit memory recall but still has the ability to maintain his own vital signs.  In this study, we design a sedative technique for bronchoscopy, a BIS-guided propofol administration, to compare with the traditional sedative technique, clinical-judged midazolam administration. Through the combination of the advantages of unique phymacokinamics of propofol and real time monitor of sedative level from BIS, we hope to provide patients undergoing bronchsocopy a more satisfied and safety sedative procedure. |
| --- |

1. Study design

| **Duration:** 2008/04 to 2011/04  Subjects: patients required elective flexible bronchoscopy or endobronchial ultrasound transbronchial biopsy.  Patient number: 500.  **Patient distribution**:  After 2% xylocaine inhalation and 5μg/kg alfentanil injection, patients were randomized into a study group: propofol infusion was titrated to keep the BIS level at 65-75 or a control group: 2mg/2min midazolam was given by clinical judgment to achieve conscious sedation.  **Evaluation and statistics**  Primary outcomes:   1. The satisfaction about bronchoscopy. 2. The incidence of complication during procedure.   Secondary outcomes:   1. Induction time, and recovery time. 2. Incidences of bronchoscopic relative symptoms.   Methods:  Study arm: BIS-guided propofol infusion  In the study group, induction was started using alfentanil 4~5μg/kg bolus following repeated propofol boluses (0.5~1.5 mg/kg) until the BIS level reached 70. During maintenance, propofol infusion (3~12 mg/kg/hour) was given using a syringe pump (Injectomat Agilia, Fresenius Kabi, France), which was titrated to keep the BIS level between 65 and 75.  Comparator arm: Clinical-judged midazolam administration  In the control group, induction was started using alfentanil 4~5μg/kg bolus following 2 mg midazolam bolus. After 2 minutes, if the patient was not well sedated, midazolam boluses were repeat by increments of 2 mg/2min until conscious sedation was achieved  **Data analysis**  Patients would score the tolerance of procedure-related symptoms by 10-point verbal analogue scale (VAS) and their willing to repeated BF if indicated. Bronchoscopists would evaluate patient cooperation by if procedure interference by patients’ movement or cough. Adverse events, sedative and procedure parameters were analyzed. |
| --- |

計畫主持人簽名：林定佑 　日期：ˍˍˍˍˍ

協同主持人簽名：羅友倫 　日期：ˍˍˍˍˍ
